# BMC Pediatrics reviewer acknowledgement 2014

**DOI:** 10.1186/s12887-015-0319-6

**Published:** 2015-03-04

**Authors:** Nawsheen Boodhun

**Affiliations:** BioMed Central, Floor 6, 236 Gray’s Inn Road, London, WC1X 8HB UK

## Abstract

The editors of *BMC Pediatrics* would like to thank all our reviewers who have contributed to the journal in Volume 14 (2014).

**Ellen Aartun**

Denmark

**Arturo Abdelnour**

Costa Rica

**Alaa Abd-Elsayed**

Egypt

**Yehia Abed**

Palestinian Territory

**Olumide Abiodun**

Nigeria

**Thomas Abramo**

USA

**Arianna Aceti**

Italy

**Eleri Adams**

UK

**Olusegun Adebami**

Nigeria

**Olufemi Adelowo**

Nigeria

**Miriam Adhikari**

South Africa

**Kingsley Agho**

Australia

**Kareem Airede**

Nigeria

**Akinyele Akinlade**

Nigeria

**Benjamin Albert**

New Zealand

**Luis M. Alegre**

Spain

**Ute Alexy**

Germany

**Khalid Alfaleh**

Saudi Arabia

**Sofiya Alhassan**

USA

**Paul Aljabar**

UK

**Bernt Alm**

Sweden

**Nicole Almenrader**

Italy

**Suzana Almoosawi**

UK

**Harith Al-Qazaz**

Malaysia

**Ghada Al-Rawahi**

Canada

**Jane Alsweiler**

New Zealand

**Teatske Altenburg**

Netherlands

**Jose Ramon Alvero Cruz**

Spain

**Luciano Alves Favorito**

Brazil

**Teresa Amaral**

Portugal

**Sergio Amarri**

Italy

**Geoffrey Ambler**

Australia

**Niels Holmark Andersen**

Denmark

**Satinder Aneja**

India

**Francois Angoulvant**

France

**Vincenzo Antona**

Italy

**Mitsuhiro Aoki**

Japan

**Christian Apfelbacher**

Germany

**Tomoki Arichi**

UK

**Tadashi Ariga**

Japan

**Emmanuel Arinaitwe**

Uganda

**Stephen Aronoff**

USA

**Pavai Arunachalam**

India

**Akira Ashida**

Japan

**Ambika Ashraf**

USA

**Greg Atkinson**

UK

**Stephanie Atkinson**

Canada

**Lamidi Isah Audu**

Nigeria

**Linda Aurpibul**

Thailand

**Inge Axelsson**

Sweden

**Eva Babusikova**

Slovakia

**Paluku Bahwere**

Malawi

**Gianluca Baio**

UK

**Muideen Bakare**

Nigeria

**Dian Baker**

USA

**Gareth Ball**

UK

**Quentin Ballouhey**

France

**Teresa Bandeira**

Portugal

**Robert Bandsma**

Canada

**Gerard Banez**

USA

**Sarfaraz Banglawala**

Canada

**Salvatore Barberi**

Italy

**Jane Barlow**

UK

**Michaela Barnard**

UK

**Jacqueline Barnes**

UK

**Benjamin Bar-Oz**

Israel

**Maru Barrera**

Canada

**Mauro Barros**

Brazil

**Michelle Barton-Forbes**

Canada

**Renato Bassan**

Italy

**Diego Garcia Bassani**

Canada

**Anna Basu**

UK

**Vijay Batra**

India

**Nerissa Bauer**

USA

**Consuelo Beck-Sague**

USA

**Lauren Becton**

USA

**Daniel Belsky**

USA

**Andrew Bennie**

Australia

**Nina Berentzen**

Netherlands

**Janet Berrington**

UK

**Gerard Berry**

USA

**Silvano Bertelloni**

Italy

**Liene Bervoets**

Belgium

**Marcel Betsch**

USA

**Ramesh Bhandari**

Nepal

**Jatinder Bhatia**

USA

**Sandeep Bhattacharya**

India

**Rahul Bhola**

USA

**Vinod Bhutani**

USA

**Sibhatu Biadgilign**

Ethiopia

**Sarah Biggs**

Australia

**Helen Binns**

USA

**Tracey Bjorkman**

Australia

**Clancy Blair**

USA

**Cynthia Blanco**

USA

**Jonathan Blau**

USA

**James Boardman**

UK

**Michael Boivin**

USA

**Srinivas Bolisetty**

Australia

**Attilio Boner**

Italy

**Maria Bonsignore**

Italy

**Erika Borkoles**

Australia

**Cynthia Boschi Pinto**

Switzerland

**Nancy Bostock**

UK

**Jérémie Botton**

France

**Marielle Karine Bouyou-Akotet**

Gabon

**Mauro Bozzola**

Italy

**Marek Brabec**

Czech Republic

**Anneke Brand**

Netherlands

**Paul Brand**

Netherlands

**Rollin Brant**

Canada

**Hilde Kristin Brekke**

Sweden

**John F P Bridges**

USA

**Shobha Broor**

India

**Jason Brophy**

Canada

**Patrizia Bruzzi**

Italy

**Joseph Buckhalt**

USA

**Christoph Buhrer**

Germany

**James Bunn**

UK

**Ian Burgess**

UK

**Scott Burgess**

Australia

**Matthew Bush**

USA

**Alison Buttenheim**

USA

**Bert Callewaert**

Belgium

**Claudia Calogero**

Italy

**Rosa Calvo**

Spain

**Cecile Cames**

France

**Roque Cardona-Hernandez**

Spain

**John Carlin**

Australia

**Magda Carneiro**

Brazil

**Genny Carrillo Zuniga**

USA

**Marco Caruselli**

France

**Sandra Carvalho-Bos**

Portugal

**Filip Cecka**

Czech Republic

**Carolina Chamorro-Vina**

Canada

**Alice Charach**

Canada

**Hassib Chehade**

Switzerland

**Wassim Chemaitilly**

USA

**Chung-Ming Chen**

Taiwan

**Shaojie Chen**

China

**Shawn Chen**

Taiwan

**Andreas Chiabi**

Cameroon

**Palma Chillón**

Spain

**Yitsiew Chin**

Malaysia

**Virginia Chomitz**

USA

**Voranush Chongsrisawat**

Thailand

**Hokyung Choung**

South Korea

**Rolando Cimaz**

Italy

**Emma Clark**

UK

**Cheril Clarson**

Canada

**Tine Dalsgaard Clausen**

Denmark

**Meryl Cohen**

USA

**Eyal Cohen**

Canada

**Conrad Cole**

USA

**R. Thomas Collins Ii**

USA

**Erica Cook**

UK

**Wouter Cools**

Belgium

**Peter Cooper**

South Africa

**Antonio Coppola**

Italy

**Giangennaro Coppola**

Italy

**E Corpeleijn**

Netherlands

**Iuri Corsini**

Italy

**Giuliana Cortese**

Italy

**Luciane Costa**

Brazil

**Malik Coulibaly**

Burkina Faso

**Charles Court-Brown**

UK

**Sherry Courtney**

USA

**Imelda Coyne**

Ireland

**Lisa Croen**

USA

**Estefania Custodio**

Spain

**Ron Dagan**

Israel

**Noémi Dahan-Oliel**

Canada

**Tuba Dal**

Turkey

**Marc Dalecki**

Canada

**Theresa Dall Helth**

Denmark

**Diane Damiano**

USA

**Carlo Dani**

Italy

**M. Carolina Danovaro-Holliday**

USA

**Peter Dargaville**

Australia

**Anne-Sophie Darlington**

UK

**Eugene Kofuor Maafo Darteh**

Ghana

**Jai Das**

Pakistan

**Luca D'Ascanio**

Italy

**Reza Daugherty**

USA

**Sean Davies**

USA

**Andrew Day**

Australia

**Gianpaolo De Filippo**

France

**Elena De La Serna**

Spain

**Peter De Winter**

Netherlands

**Maartje De Wit**

Netherlands

**Iyabode Olabisi Dedeke**

Nigeria

**Seyed Mohsen Dehghani**

Iran

**Mario D'Elios**

Italy

**Maurizio Delvecchio**

Italy

**Eugene Dempsey**

Ireland

**Ankita Desai**

USA

**Ulrich Desselberger**

UK

**Angelica Dessì**

Italy

**Kassahun Desta**

Ethiopia

**Anna Rita Di Biase**

Italy

**Graziella Di Grezia**

Italy

**Jose Luis Diaz-Rossello**

Uruguay

**Robert Digeronimo**

USA

**Sharmila Dissanaike**

USA

**Hazel Dockrell**

UK

**Nora Doering**

Sweden

**Monika Dos Santos**

South Africa

**Marjan Drukker**

Netherlands

**Jennifer Duchon**

USA

**Sourabh Dutta**

India

**Leigh Dyet**

UK

**Kevin Dysart**

USA

**Eric Dziuban**

USA

**Narelle Eather**

Australia

**Liza Edmonds**

New Zealand

**Matthias Egri-Okwaji**

Nigeria

**Christopher Bismarck Eke**

Nigeria

**Ashraf El Metwally**

UK

**Ann-Christin Eliasson**

Sweden

**Heather Elphick**

UK

**Nina Emaus**

Norway

**Stephen Eppes**

USA

**Omer Erdeve**

Turkey

**Melinda Erdos**

Hungary

**Esperanza Escortell**

Spain

**Christopher Imokhuede Esezobor**

Nigeria

**Sandrine Essouri**

France

**Irene Esteban-Cornejo**

Spain

**Charlotte Evans**

UK

**Jessica Ezri**

Switzerland

**Iretiola Fajolu**

Nigeria

**Giacomo Faldella**

Italy

**Kathryn Farrow**

USA

**Alan Farrow-Gillespie**

USA

**Moncef Feki**

Tunisia

**Juan Miguel Fernandez-Alvira**

Spain

**Bolanle Fetuga**

Nigeria

**Josep Figueras-Aloy**

Spain

**Peter Filan**

Ireland

**Martijn Finken**

Netherlands

**Emily Finne**

Germany

**Saker Firas**

USA

**Marion Flechtner-Mors**

Germany

**Janice Fletcher**

Australia

**Sue Fletcher-Watson**

UK

**Cesar G Fontecha**

Spain

**Maria Forns**

Spain

**Terrence Forrester**

Jamaica

**Nathan Fosse**

USA

**Sotirios Fouzas**

Greece

**Lawrence Foweather**

UK

**Gustavo Fraga**

Brazil

**Adriana Franco**

Brazil

**Inge Franki**

Belgium

**David Freedman**

USA

**Helen Fryssira**

Greece

**Susan Fuchs**

USA

**Yuki Fujita**

Japan

**Ryuji Fukazawa**

Japan

**Jayne Fulkerson**

USA

**Daniel Fung**

Singapore

**Christoph Fusch**

Canada

**Dario Galante**

Italy

**Alexander Gallus**

Australia

**Stacey Galowitz**

USA

**Livia Garavelli**

Italy

**Anil Garg**

UK

**Orsolya Genzel**

Germany

**T Gheita**

Egypt

**Maria Gianni'**

Italy

**Peter Gerard Gibson**

Australia

**Stefania Giedrys-Kalemba**

Poland

**Cathleen Gillespie**

USA

**Flavio Giordano**

Italy

**John Giuliano**

USA

**Frances Glascoe**

USA

**Saralee Glasser**

Israel

**Sarah Goff**

USA

**Jeanne Goldberg**

USA

**David Goldfarb**

Canada

**Andrea Goldschmidt**

USA

**Vivianne Calheiros Chaves Gomes**

Brazil

**Antonia Gómez Conesa**

Spain

**Fangqi Gong**

China

**Timothy Goodacre**

UK

**Andy Goren**

USA

**Paul Govaert**

Belgium

**Monika Goyal**

USA

**Stephen Graham**

Australia

**Serge Grazioli**

Switzerland

**Christopher Greeley**

USA

**Siri Atma Greeley**

USA

**Ian Griffin**

USA

**Paul Gringras**

UK

**Floris Groenendaal**

Netherlands

**Giuseppe Grosso**

Italy

**Paulo Guerra**

Brazil

**Mintaze Gunel**

Turkey

**Ingibjorg Gunnarsdottir**

Iceland

**Rakesh Gupta**

Nepal

**Samir Gupta**

UK

**Sarika Gupta**

India

**Julie Gutman**

USA

**Gerda-Maria Haas**

Germany

**Karen Hacker**

USA

**Wendy Hall**

Canada

**Michele Hamm**

Canada

**Branka Hanicar**

Croatia

**Iain Hargreaves**

UK

**Deirdre Harrington**

UK

**Jo Hart**

UK

**Annelies Hartman**

Netherlands

**Mohammad Hasan**

Qatar

**Ferdinand Haschke**

Austria

**Jean-Michel Hascoet**

France

**Amanda Hassinger**

USA

**Bryan Haughom**

USA

**Michael Hawkes**

Canada

**Shogo Hayashi**

Japan

**Nicole Hayde**

USA

**Meizi He**

USA

**Nongyue He**

China

**Xiangui He**

China

**Lana Hebden**

Australia

**Berit Heitmann**

Denmark

**Onno Helder**

Netherlands

**Krista Helleman**

Canada

**Nicholas Henschke**

Germany

**Michael Hermanussen**

Germany

**Kenneth Herrmann**

USA

**Egbert Herting**

Germany

**Ann Hickey**

UK

**Matthew Hicks**

Canada

**Takahiro Higashi**

Japan

**Marisa Hilliard**

USA

**Robert Hilliard**

Canada

**Trina Hinkley**

Australia

**Gerrit Hirschfeld**

Germany

**Jill Hnatiuk**

Australia

**Andrew Hodge**

Australia

**Falk Hoffmann**

Germany

**Rebecca Hommer**

USA

**Brian Hopkins**

UK

**Mary Horan**

Ireland

**Mina Hosseinipour**

Malawi

**Erin Howie**

Australia

**Yu-Shu Huang**

Taiwan

**Li Hui**

China

**Catherine Hunter**

USA

**Murad Husein**

Canada

**John Hutson**

Australia

**Arnold Huurnink**

Netherlands

**Bede Ibe**

Nigeria

**Aamer Imdad**

USA

**Marita Rohr Inglehart**

USA

**Moshe Ipp**

Canada

**Mary Irvine**

USA

**Iman Iskander**

Egypt

**Saurabh Jain**

UK

**Tim Jaspan**

UK

**Jennifer Margaret Jelsma**

South Africa

**Andreas Jenke**

Germany

**Ruth Jepson**

UK

**Milos Jesenak**

Slovakia

**Julie Jesson**

France

**Gerda Jimmy**

Switzerland

**Lars Jødal**

Denmark

**William Johnson**

UK

**Christine Jones**

UK

**Kelsey Jones**

UK

**Peter Jones**

UK

**Björn Jonsson**

Sweden

**Shashank Joshi**

India

**Rumaya Juhari**

Malaysia

**Madarina Julia**

Indonesia

**Camille Jung**

France

**Joanna Jurewicz**

Poland

**Janine Jurkowski**

USA

**Masaharu Kagawa**

Japan

**Benjamin Kagina**

South Africa

**David Kalfa**

USA

**Satish Kalhan**

USA

**Hee Gyung Kang**

South Korea

**Henry J Kaplan**

USA

**Anne-Marie Kappelgaard**

Denmark

**Tonya Kara**

New Zealand

**Walter Karlen**

Switzerland

**Kianoush Kashani**

USA

**Lakshmi Katakam**

USA

**Anup Katheria**

USA

**Han Cg Kemper**

Netherlands

**Jorien Kerstjens**

Netherlands

**Anuradha Khadilkar**

India

**Jaroslaw Kierkus**

Poland

**Jinkwan Kim**

South Korea

**Youngwon Kim**

USA

**Russell Kirby**

USA

**Gil Klinger**

Israel

**Martin Kneyber**

Netherlands

**Anders Koch**

Denmark

**Henrik Koehler**

Germany

**Anastasios Kollias**

Greece

**Joe Kossowsky**

USA

**Michael Kramer**

Canada

**Nancy Krebs**

USA

**Elena Krumova**

Germany

**Haytham Kubba**

UK

**Chandrakanta Kumar**

India

**Surender Kumar**

India

**Rashmi Kumar**

India

**Vishwajeet Kumar**

India

**Vasanth Kumar**

USA

**Ho-Chang Kuo**

Taiwan

**Ozgur Kurt**

Turkey

**Soyang Kwon**

USA

**Harrie Lafeber**

Netherlands

**Rajalakshmi Lakshman**

UK

**Veronica Lambert**

Ireland

**Steven Lamm**

USA

**Amy Lampard**

USA

**Claudio F. Lanata**

Peru

**Jason Lang**

USA

**Thorsten Langer**

Germany

**Malinee Laopaiboon**

Thailand

**Costas Laparidis**

Greece

**Alexandre Lapillonne**

France

**David P. Laplante**

Canada

**Antoinette Laskey**

USA

**Annie Lau**

Australia

**Elise Launay**

France

**Joshua Lawson**

Canada

**Kirsty Le Doare**

South Africa

**Nicole Le Saux**

Canada

**Wang-Tso Lee**

Taiwan

**Hung-Chang Lee**

Taiwan

**Pamela Lee**

Vatican City State

**Traci Leong**

USA

**Ting Fan Leung**

China

**Ting Fan Leung**

Hong Kong

**Orly Levit**

USA

**Richard Lewanczuk**

Canada

**Albert Li**

Hong Kong

**Lun-De Liao**

Singapore

**Jean-Michel Liet**

France

**Choon Guan Lim**

Singapore

**Ming Lim**

UK

**Hung-Chih Lin**

Taiwan

**Ying-Jui Lin**

Taiwan

**Ornella Lincetto**

Italy

**Sergio Line**

Brazil

**Alexandre Linhares**

Brazil

**Yoav Littner**

USA

**Shuwei Liu**

China

**Rong Liu**

China

**Robert Locke**

USA

**Rakesh Lodha**

India

**Deirdre Logan**

USA

**Jamie Lohr**

USA

**Sebastian Loos**

Germany

**Armando Lorenzo**

Canada

**Linda Lowes**

USA

**Kelly Lowry**

USA

**Jeffrey Lu**

USA

**Laura Lucaccioni**

Italy

**Marta Lucchetta**

Italy

**Julie Lumeng**

USA

**Noni Macdonald**

Canada

**Aristides Machado-Rodrigues**

Portugal

**Michelle Macias**

USA

**Maciste Macias-Cervantes**

Mexico

**Andrea Maciel-Guerra**

Brazil

**Helen Mactier**

UK

**Jagmeet Madan**

India

**Agnieszka Madej-Pilarczyk**

Poland

**K S Madhusudhan**

India

**Jose Maia**

Portugal

**Nunzia Maiello**

Italy

**Imad Makhoul**

Israel

**Conor Mallucci**

UK

**Marisa Mancini**

Brazil

**Cedric Manlhiot**

Canada

**Jacob Manteuffel**

USA

**Paola Marchisio**

Italy

**Carole Marcus**

USA

**Dean Markic**

Croatia

**Jessica Markowitz**

USA

**Stephen Markwell**

USA

**Pedro Marques-Vidal**

Switzerland

**Stefano Marventano**

Italy

**Harald Matthes**

Germany

**Inas Mazen**

Egypt

**Mumtaz M Mazicioglu**

Turkey

**Graham Mccaffrey**

Canada

**H. David Mccarthy**

UK

**Eric Mccollum**

Malawi

**Nichola Mccullough**

UK

**Jenny Mcdonald**

Australia

**Eleanor Mcgee**

UK

**Meghan Mcgrady**

USA

**Patricia Mckane**

USA

**Ruth Mckee**

UK

**Stephen Edward Mcmillin**

USA

**Catherine Mcneal**

USA

**Alan Mendelsohn**

USA

**Jucille Meneses**

Brazil

**Christine Metz**

USA

**Fabrice Michel**

France

**Karen Mickle**

Australia

**Gregorio Paolo Milani**

Italy

**Christophe Milesi**

France

**Jonathan Miller**

USA

**Irene Milliken**

UK

**Matthieu Million**

France

**Jonathan Mintzer**

USA

**Rebecca Mitchell**

Australia

**Ron Mitchell**

USA

**Maurice B Mittelmark**

Norway

**Anup Mohta**

India

**Rachel Moon**

USA

**Laura Moore**

USA

**Dewton Moraes Vasconcelos**

Brazil

**Iris Morag**

Israel

**Fernando Moraga**

Bangladesh

**Alina Morawska**

Australia

**Henner Morbach**

Germany

**Philip Morgan**

Australia

**Prue Morgan**

Australia

**Ichiro Morioka**

Japan

**Michela Jane Angela Morleo**

UK

**Kevin Morris**

UK

**Rustin Morse**

USA

**Fabio Mosca**

Italy

**Anja Mowes**

USA

**Michael Msall**

USA

**Holger Muehlan**

Germany

**Noor Muhammad**

Germany

**Aleixo Muise**

Canada

**Luke Mullany**

USA

**Saul Mullen**

Australia

**Jan Müller**

Germany

**Caroline Mulvaney**

UK

**Antonella Muraro**

Italy

**Muhammad Umair Mushtaq**

Pakistan

**Victor Musiime**

Uganda

**Samridh Nagar**

Australia

**Koichi Nakanishi**

Japan

**Takahiro Nakayama**

Japan

**Gertrude Namazzi**

Uganda

**Nader Nassif**

Italy

**Nagato Natsume**

Japan

**Ellis Neufeld**

USA

**Mark Neuman**

USA

**Jason Newland**

USA

**Daniel Ng**

Hong Kong

**Glen Nielsen**

Denmark

**Signe Smith Nielsen**

Denmark

**Susan Niermeyer**

USA

**Fidelis Njokanma**

Nigeria

**Iona Novak**

Australia

**Anita Nucci**

USA

**Irena Nulman**

Canada

**Bright Nwaru**

UK

**Ju Lee Oei**

Australia

**Tinuade Ogunlesi**

Nigeria

**April Oh**

USA

**Bisola Ojikutu**

USA

**Ayodele Ojuawo**

Nigeria

**John Okeniyi**

Nigeria

**Julicristie Oliveira**

Brazil

**Laura Olivieri**

USA

**Olaniyi Onayemi**

Nigeria

**Mehmet Yekta Oncel**

Turkey

**Yoon Phaik Ooi**

Switzerland

**Saheed Oseni**

Nigeria

**Michael O'Shea**

USA

**Kevin Osterhoudt**

USA

**Jun Oto**

Japan

**Fengxiu Ouyang**

China

**Nina Cecilie Overby**

Norway

**Joshua Owa**

Nigeria

**Olusola Oyedeji**

Nigeria

**Verity Pacey**

Australia

**Dasja Pajkrt**

Netherlands

**Deepak Palakshappa**

USA

**Ginny Paleg**

USA

**Arto Palmu**

Finland

**Stavroula Papapdodima**

Greece

**Karthikeyan Paranthaman**

UK

**Jae Park**

USA

**Marzia Pasquali**

USA

**Maria Francesca Patria**

Italy

**David Paul**

USA

**Malgorzata Paul**

Poland

**Stephen Pearlman**

USA

**Natalie Pearson**

UK

**Lydia Pecker**

USA

**Dino Pedrotti**

Italy

**Giles Peek**

UK

**Suzanne Penfold**

Slovakia

**Suzanne Penfold**

UK

**Ji-Bin Peng**

USA

**Supa Pengpid**

Thailand

**Marco Peres**

Australia

**Jeffrey Pernica**

Canada

**Roberto Perniola**

Italy

**Cynthia Peterson**

Switzerland

**Laetitia-Marie Petit**

Switzerland

**Anne Pham-Huy**

Canada

**Terri Pikora**

Australia

**Daniele Piovani**

Italy

**Maryam Piram**

France

**Paul L. Plener**

Germany

**Christian Poets**

Germany

**Marko Pokorn**

Slovenia

**Aziz Polat**

Turkey

**Alexey Polonikov**

Russian Federation

**George Porter**

USA

**Anna Price**

Australia

**Joseph Price**

USA

**Jochen Profit**

USA

**Evan Propst**

Canada

**Jeremy Pryce**

UK

**John William Lambert Puntis**

UK

**Mikulas Pura**

Slovakia

**Niina Puustinen**

Finland

**Shamim Qazi**

Switzerland

**Liqian Qiu**

China

**Jon Quach**

Australia

**José M Quintillá**

Spain

**Alejandro Quiroga**

USA

**Omer Qutaiba B. Allela**

Iraq

**Jerilynn Radcliffe**

USA

**Nedeljko Radlovic**

Serbia

**Melanie Rank**

Germany

**Susanna Ranta**

Sweden

**Rakesh Rao**

USA

**David Rappaport**

USA

**Sabrina Rasheed**

Bangladesh

**Maggie Redshaw**

UK

**Christopher Alan Reid**

Australia

**Aiguo Ren**

China

**James Renner**

Nigeria

**Bernhard Resch**

Austria

**Emrush Rexhaj**

Switzerland

**Corsino Rey**

Spain

**Zaccaria Ricci**

Italy

**Nicola Diane Ridgers**

Australia

**Marshall Riley**

UK

**Arieh Riskin**

Israel

**Calum Roberts**

Australia

**Joan Robinson**

Canada

**Elena Rodriguez-Rodriguez**

Spain

**Charles Christoph Roehr**

UK

**Martin Roland**

UK

**Nigel Rollins**

South Africa

**Diane Roscoe**

Canada

**Stephen Rose**

Australia

**Rebecca Rudd**

USA

**Franca Rusconi**

Italy

**Kelli Ryckman**

USA

**Carla Sabariego**

Germany

**Matthew Sabin**

Australia

**Manish Sadarangani**

Canada

**Raphael Saginur**

Canada

**Pilar Sainz De Baranda**

Spain

**Rie Sakai**

USA

**Leanne Sakzewski**

Australia

**Katherine Salamon**

USA

**Silvia Regina Saldiva**

Brazil

**Lucia Salesi**

Italy

**Shamiel Salie**

South Africa

**Vanessa Sanchez-Gistau**

Spain

**Francesca Santamaria**

Italy

**Sergio Santos**

Brazil

**Laura Sauve**

Canada

**Lindsay Schenkel**

USA

**Peter Schmidt**

Australia

**Lourdes Schnaas**

Mexico

**Sven M Schulzke**

Switzerland

**Jane Scott**

Australia

**Shannon Scott**

Canada

**André Seabra**

Portugal

**Jan Seghers**

Belgium

**Kris Sekar**

USA

**Ben Semmekrot**

Netherlands

**Moinak Sen Sarma**

India

**Jeremiah Seni**

Tanzania

**Ali Sepahdari**

USA

**Mary Serdula**

USA

**Raju Shah**

India

**Sameena Shah**

Pakistan

**Ketan Shankardass**

Canada

**Iman Sharif**

USA

**Eesha Sharma**

India

**Yiping Shen**

USA

**Xiaoyang Sheng**

China

**Patricia Shewokis**

USA

**Ling Shi**

USA

**Zhongjie Shi**

USA

**Jung Yeon Shim**

South Korea

**Masaki Shimizu**

Japan

**Kevin Short**

USA

**Shyh-Dar Shyur**

Taiwan

**Loreana Silveira**

Brazil

**Aryeh Simmonds**

Israel

**Ajay Sinha**

UK

**Susan Sisson**

USA

**Seter Siziya**

Zambia

**Joseph Skelton**

USA

**Marianne Skreden**

Norway

**Linda Slack-Smith**

Australia

**Tina Slusher**

Nigeria

**Françoise Smets**

Belgium

**Claire Smith**

UK

**Lucy Smith**

UK

**Joern Soltau**

USA

**Madhuri Sopirala**

USA

**Maroje Soric**

Croatia

**Rick Speare**

Australia

**Carsten Speckmann**

Germany

**Jan Sperhake**

Germany

**Claudio Spinelli**

Italy

**Jane Squires**

USA

**Vijay Srinivasan**

USA

**Keith St Lawrence**

Canada

**Philippe Steenhout**

Switzerland

**John Stefano**

USA

**Sarah Stewart-Brown**

UK

**Blanka Stiburkova**

Czech Republic

**John F. Stins**

Netherlands

**Chris Stockmann**

USA

**Jana Strahler**

Germany

**Robyn Stremler**

Canada

**Wendy Sturtz**

USA

**Adewale Okanlawon Sule-Odu**

Nigeria

**Kevin Sullivan**

USA

**Carolyn Summerbell**

UK

**Xin Sun**

China

**Sharon Sung**

Singapore

**Jc Suris**

Switzerland

**Gordana Susic**

Serbia

**Graeme Suthers**

Australia

**Elisabeth Svensson**

Sweden

**Meenakshi Swaminathan**

India

**David Sweet**

UK

**Dorota Szostak-Wegierek**

Poland

**Taro Takeshima**

Japan

**Ajay Talati**

USA

**Alison Talbert**

Kenya

**Pooja Tandon**

USA

**Patrick Tang**

Canada

**Jose Tapia**

Chile

**Kimberly Tartaglia**

USA

**Richard Telford**

Australia

**Roger Thomas**

Canada

**Pam Thomason**

Australia

**Dana Thompson**

USA

**Michael Thompson**

USA

**Dick Tibboel**

Netherlands

**Alison Tigg**

Australia

**Janice Tijssen**

Canada

**Peter Tilley**

Canada

**Ariel Toloza**

Argentina

**Olukemi Tongo**

Nigeria

**Karina Top**

Canada

**Susan B Torrey**

USA

**Antonella Tosti**

USA

**Leonardo Trasande**

USA

**Victoria Trenchs**

Spain

**Antonia Trichopoulou**

Greece

**Chang-Yong Tsao**

USA

**Takeshi Tsutsumi**

Japan

**Michael Tunik**

USA

**Jos Twisk**

Netherlands

**Agozie Ubesie**

Nigeria

**Raphael Udassin**

Israel

**Holm Uhlig**

UK

**Nicola Ullmann**

Italy

**Michael Urschitz**

Germany

**Nestor Vain**

Argentina

**Christina Valentine**

USA

**Monique Van De Lagemaat**

Netherlands

**Hubertus Van Hedel**

Switzerland

**Wendy Van Lippevelde**

Belgium

**Femke Van Nassau**

Netherlands

**Frits Van Rhee**

USA

**Diny Van Zoeren-Grobben**

Netherlands

**Jillon Vander Wal**

USA

**Leigh Vanderloo**

Canada

**Patrick Vandevoorde**

Belgium

**George Vaos**

Greece

**Ivan Varga**

Slovakia

**Atul Vats**

USA

**Timothy Vece**

USA

**Pierangelo Veggiotti**

Italy

**Haritha Vellanki**

USA

**Maximo Vento**

Spain

**Charles Verge**

Australia

**Nishant Verma**

India

**Rita Verma**

USA

**Ritu Verma**

USA

**Ruud Vermeulen**

Netherlands

**Sunil Vernekar**

India

**Vanessa Ann Vigilante**

USA

**Jeffrey M Vinocur**

USA

**Tommy Visscher**

Netherlands

**Carl Von Baeyer**

Canada

**Sarah Von Spiczak**

Germany

**Wieger Voskuijl**

Malawi

**Amos Vromen**

Israel

**Anne Vuillemin**

France

**Gordan Vujanic**

UK

**Julian Vyas**

New Zealand

**Julee Waldrop**

USA

**Andrew Walley**

UK

**Guoying Wang**

USA

**Martin Ward Platt**

UK

**Klaas Wardenaar**

Netherlands

**Malgorzata Wasniewska**

Italy

**Andrea Waylen**

UK

**Ashley Weaver**

USA

**Patricia Weng**

USA

**Karla Wentzel**

Canada

**Fred Were**

Kenya

**Benjamin Wheeler**

New Zealand

**Elspeth Whitby**

UK

**Chris White**

Australia

**Andrea Wickremasinghe**

USA

**Kurt Widhalm**

Austria

**Frank Wieringa**

France

**Bridget Wilcken**

Australia

**Beth Wildman**

USA

**James Wiley**

USA

**Suzanne Willard**

USA

**Paige Williams**

USA

**Jennie Wilson**

UK

**Kathleen Wilson**

USA

**Deanne Wilson-Costello**

USA

**Esko Wiltshire**

New Zealand

**Yk Wing**

Hong Kong

**Corinna Winter**

Germany

**David Wirtschafter**

USA

**Cicek Wöber-Bingöl**

Austria

**Cicek Wöber-Bingöl**

Germany

**Joseph Wolfsdorf**

USA

**Katja Wolthers**

Netherlands

**Craig Wong**

USA

**Melanie Wong**

Australia

**Kyung In Woo**

South Korea

**Jayne Woodside**

UK

**Charlotte Wright**

UK

**Julie Wright**

USA

**Robert Wright**

USA

**Rebecca Wyse**

Australia

**Yao-Jong Yang**

Taiwan

**Allen Eng Juh Yeoh**

Singapore

**Ediz Yesilkaya**

Turkey

**Bridget Young**

UK

**Jeanine Young**

Australia

**Zhangbin Yu**

China

**Justin Zachariah**

USA

**Samuel Zangen**

Israel

**Huan Zeng**

China

**Jihui Zhang**

Hong Kong

**Nan Zhang**

UK

**Linjie Zhang**

Brazil

**Dp Zhao**

China

**Lili Zhao**

USA

**Guangwei Zhou**

USA

**Ying Zhou**

USA

**Jiajun Zhu**

China

**Vahid Ziaee**

Iran

**Joseph Zickafoose**

USA

**Jerry Zimmerman**

USA

**Maddalena Zippi**

Italy

**Alvin Zipursky**

Canada

**Elizabeta Zisovska**

Macedonia

**Anna Zolotnitskaya**

USA

**Gian Vincenzo Zuccotti**

Italy

**Rose Zulliger**

USA

**Jill Zwicker**

Canada

